# Microbiome in the urinary system—a review

**DOI:** 10.3934/microbiol.2017.2.143

**Published:** 2017-03-20

**Authors:** Jane Tang

**Affiliations:** National Security and Intelligence, Noblis, Reston, VA, USA

**Keywords:** microbiome, urinary system, microbiota, urinary tract infection, urinary microbiome

## Abstract

Urine was considered sterile in healthy individuals for many years, and the presence of bacteria signified urinary tract infection. With the development of Expanded Quantitative Urine Culture (EQUC) and utilization of molecular techniques, the previous clinical dogma is no longer valid. Instead, healthy people harbor a considerable microbial community, or microbiota, in their urinary systems. Similar to other physiological niches where microbiota contribute to the health status of their hosts, recent studies demonstrated different microbial populations also play a crucial role in urinary health of individuals. Understanding urinary microbiome thus allows a more holistic approach in the diagnosis, treatment, and prevention of diseases and disorders in urinary system. This review article provides an overview of current findings in urinary microbiome and discusses some of the gaps for future research.

## 1. Introduction

The Human Microbiome Project (HMP) launched in 2008 ushered in a new era of understanding the microbial communities residing with us. Until then, we did not realize that microorganisms outnumbered our cells by tenfold, and a great majority were unknown because they have yet to be cultured and studied [1]. The HMP enabled numerous results and insights into the intricate and important roles these microbes play in our health, ranging from metabolism and phenotype modulation (such as obesity), innate immune system development, to etiology for diseases or conditions (due to microbial ecosystem imbalance) [2],[3]. New findings challenge our current thinking and approaches, and they prompt paradigm shift towards alternative solutions in disease diagnosis, prevention, and treatments.

The HMP focused on five sites: gastrointestinal, oral, skin, nasal and urogenital. Urinary system was not part of the study because traditionally urine was considered sterile in healthy individuals [4]–[8]. This observation dated back to the mid 1800's when microbiology was still in its infancy and it continued to be upheld based on bacterial culturing outcomes. The clinical protocol and conditions for detection, however, only yield limited number and types of bacteria [4]. In the last few years with advanced culturing techniques and molecular approaches, we realized the urinary system contains extensive numbers and members of bacteria in healthy and asymptomatic individuals [9]–[12]. They are uniquely different than populations in the gut and vagina, two niches in close proximity from the bladder [5]. Their roles as urinary microbiota in maintaining our health and predisposing to a variety of diseases and disorders are just beginning to be elucidated [4],[5],[8],[13],[14].

This review provides a general overview of urinary microbiome, its compositions and associations with different age and gender. The effects of these microbes in the health status are further illustrated by examples of diseases and disorders resulted from changes of microbial communities. The paper concludes with research gaps and future directions of urinary microbiome research.

## 2. Microbiome in the Urinary System

### 2.1. Urine is not sterile

The clinical dogma, up to very recently, assumed urine from healthy individuals was sterile and the presence of bacteria signified some type of infection in the urinary tract [6]. As a body fluid, urine is hostile to the survival of microorganisms. It is hypertonic with low pH (average around pH 6), contains high concentration of urea which is inhibitory to most bacteria, and other substances including some that possess antimicrobial properties [13]. Additionally, the frequent flushing and voiding make persisting in the bladder difficult. Those that can survive, colonize and cause infection are termed uropathogens, and they often possess virulence factors such as host cell attachments, biofilm formation, host defense evasion, and antimicrobial resistance development [15]. Additional mechanisms to survive in this harsh environment include utilizing urine as substrate for growth, upregulating genes involved in iron transport, and devising systems to adapt in low pH, high urea concentrations and hypertonic environments [13].

Another contributing factor to the long acceptance of urine as sterile was the clinical procedure for detecting bacteria in urine samples. Since the 1950's the gold standard for confirming urinary tract infection (UTI) has been the urine culture. Over the years, this detection method has proven questionable because the urine culture conditions are very restrictive, thus limits in the types of bacteria that are recoverable. The protocol specifies a very small amount of sample (1 µl of urine), plated only on two microbiological media (blood agar and MacConkey agar), and incubated at 35 °C for 24 hours. These culture conditions favor fast-growing aerobic bacteria and are designed for uropathogens such as *Escherichia (E.) coli*. Those that require special nutrient, takes more than 24 hours to grow, or prefer anaerobic environment (bladder is low in oxygen) are frequently missed from this screening [8],[11],[12],[16]. Furthermore, the threshold to indicate a positive urine culture has been debated and changed throughout the years [17]. In general, 10^5^ colony forming units (CFU)/mL indicate UTI; however, some urinary disorders resulted from lower number of organisms (<10^3^ CFU/mL) are overlooked by this routine culture method [4],[10],[16],[17].

The advance of molecular techniques enabled a more comprehensive assessment of the microbes present in urine. The 16S ribosomal RNA gene sequence analysis yielded different types of bacteria in healthy urine [4],[5],[9],[10]. In fact, the bladder may include resident bacteria flora such as *Lactobacillus*, *Staphylococcus*, *Gardnerella*, just to name a few [11],[12],[18]. One study reported a total of 321 genera from bladders of culture-negative women [12]. The disparity between culture and molecular assay prompted the development of Expanded Quantitative Urine Culture (EQUC) [10]. It uses more sample (10 µL or 100 µL) to plate on a variety of media, and incubate under different temperatures and atmospheric conditions [17],[19]. A comparison between the clinical microbiology laboratory protocol and EQUC showed 90% false-negative rate for the former gold standard and missed 67% uropathogens overall [10],[18],[19],[20]. Additionally, EQUC helps to determine many of the organisms identified by 16S rRNA gene sequencing are viable and cultivable under the appropriate growth conditions [4],[7],[10],[20]. These findings led to the recommendation of a “streamlined EQUC” as a supplemental test for individuals suspected of UTI but had negative standard urine culture [19].

These emerging evidences dispute that healthy bladder is sterile; rather, the bacterial community found in the urinary system has implications in the health status of an individual [5],[8],[10],[11],[21]. Although urine microbiome shared many similar genera with other sites in the body, it is less diverse and lower in number when compare with microbiome found in other physiological systems such as the gut and skin [18]. Nevertheless, the urinary tract is a unique environment niche for microbes, and the new knowledge associated with urinary microbiome opens door to better understanding of urinary system diseases, including implications in obstetrics and gynecology as expressed by a clinical opinion [11].

### 2.2. Urinary microbiome among different populations

#### 2.2.1. Gender

Studies have indicated the urinary microbiome is distinctive between men and women, likely due to differences in anatomical structures and hormones [5]. In general, females have a more heterogeneous mix of bacterial population than males in their clean catch mid-stream urine samples, reflecting the bladder microbiome determined by 16S rRNA analyses [9]. One study showed significantly higher number of *E. coli* cultured from healthy adult women (91%) than men (25%) [21]. *Lactobacillus* species are the most dominant members of urine microbiome in women during child bearing age [22]. Other microbiome identified by molecular method (16S rRNA gene sequencing) in female samples included representative members of the *Actinobacteria* (e.g., *Actinomyces*, *Arthobacter*) and *Bacteroidetes* (e.g., *Bacteroides*) phyla which were absent from male samples [9]. When considering the core microbiome with varied abundance in the urinary tract throughout life, both men and women possess three predominant genera—*Lactobacillus*, *Corynebacterium*, and *Streptococcus* [9],[23],[24].

There were less reports regarding urinary microbiome in men. One study employing 16S rRNA gene sequencing to characterize bacterial communities in urine collected from adolescent men and found the predominant genera as *Streptococcus, Gardnerella, Lactobacillus* and *Veillonella* [23]. Another group investigated healthy urine microbiome in both men and women; using 16S rRNA gene sequencing and metaproteomic analyses they reported higher relative abundance of *Corynebacterium* in men [24]. This genus is considered a common superficial skin flora, and its members possibly residing in the proximal urethra and/or bladder as well as to the distal urethra. Additional findings were *Enterococcus, Proteus, Klebsiella*, and *Aerococcus* as more predominate in male urine.

Variation of urinary microbiome between females and males may also stem from the different compositions in their urine. Women produce higher amount of citrate but less calcium and oxalate, whereas males excrete more creatinine [13]. The different constituents may favor certain microbes to survive in this niche, and they in turn contribute to the health status of their hosts. For example, women are more prone to UTI mostly due to anatomical reasons. However, there are evidences indicating microbes may also play a role in the occurring and recurring of UTI (see section 3.1), and their growth is likely promoted by certain substrates and hormones present in the female urine.

#### 2.2.2. Age

Urine microbiome, similar to microbial communities in other physiological systems, changes with the individual's age [5]. Children and adults have different microbiome populations, likely due to urinary metabolites from diet change, personal hygiene and voiding habits. There are different bacterial genera within age groups among the adults [9]. Changes in hormones may have an influence in the microbiome [25]; a few studies indicated *Lactobacillus* species increased during puberty and decreased after menopause [4],[5],[19]. The microbiome could change in women who become pregnant, whereas those who are not pregnant seem to have more stable microbial compositions [22]. As one gets older, constipation and urinary incontinence may alter the survival and persistence of microbes. Symptoms of lower urinary tract seem more prevalent as people age [17]. Using molecular analyses (16S rRNA gene amplification), one study noted four genera were exclusive among the elderly (age 70+) irrespective of the gender: *Jonquetella*, *Parvimonas*, *Proteiniphilum* and *Saccharofermentans* [9]. These are not well known bacteria and only recently named. Common features among them include anaerobic and fastidious nutrient requirements which made cultivation by conventional methods difficult [26]–[29] . The implications of these organisms in the elderly urinary microbiome are still unknown and await further studies [5].

The changes in urinary microbiome during one's lifetime have been associated with different diseases, and some will be further discussed in the next section. One example is the decrease of vaginal *Lactobacillus* spp. in women after menopausal allows colonization of uropathogens, resulting in increased UTI cases [30]. It is interesting to note that recurrent UTIs are not limited to older women; in fact, it is common among young and healthy women as well [31]. There are no obvious reasons for such observation, such as identifiable risk factors or abnormal urinary tracts associated with these occurrences [31]. Another example is catheter users who are at high risk of UTI. Catheter-associated urinary tract infection is a concern for the elderly as it is a common infection in nursing homes. Nevertheless, this type of infection also affects patients in acute care regardless of age [32]. Lastly, the bacteria *Oxalobacter* *formigenes* has been demonstrated with a possible role in urolithiasis (i.e., kidney stones) [33]. It's colonization in the gut seems to be age-dependent, present in children up to 8 years old and then starts to decline by age 12 into adulthood [5]. Urolithiasis cases are higher in adults, and some postulate the lower number of *O. formigenes* along with other factors may contribute to the increased concentration of oxalate in these individuals' urine [34],[35].

## 3. Association with Diseases and Disorders

### 3.1. Urinary tract infection

As the second most common type of infection, UTI accounts for approximately 8.1 million visits to the clinics in 2012 [31],[36]. Among them women are more likely to encounter such infections due to anatomical structure (approximately 50% experience UTI in their lifetime), and the elderly with multiple risk factors (e.g., obstruction, poor hygiene, diet and fluid intake) [21]. According to the American Urological Association, an estimated 150 million UTIs occur annually worldwide costing $6 billion in health care [37]. Obviously this disease is wide spread and poses a significant burden to the financial and healthcare systems.

The UTI is commonly associated with *E. coli*, and it has been implicated in >70% uncomplicated and 25–50% complicated cases [15]. The infection is frequently persistent, and with each UTI the risk of having recurrence increases. For example among women with UTI, 20–25% will develop recurrent infection and 3% within 6 months after treatment [25]. Although *E. coli* has been considered as the main uropathogen in UTI cases, a prospective study examined individuals with chronic lower urinary tract symptoms found additional organisms associated with these illnesses. Using EQUC and 16S rRNA gene sequencing methods, species of *Enterococcus* and *Staphylococcus* were frequently recovered in urine samples regardless of collection methods (clean catch midstream or by catheters) [17]. This suggests the spectrum of bacterial colonization in the urinary tract is broader than previously thought.

There are two types of recurrent infection—relapse and reinfection. The former is caused by the same organism with a shorter interval (within 2 weeks) from the original infection. On the other hand, reinfection is considered a new episode of illness resulted from a different strain of bacteria, and each occurrence is separated by a month after antibiotic treatment [31]. These distinctions were often difficult to discern in the past; however, advanced molecular technologies for bacteria identification now enable strain differentiation which could aid in determining the type of recurrence. This suggests important clinical implications because relapsing infection may result from metabolic, functional or structural abnormalities; thus, requiring more extensive urological evaluation or longer antimicrobial therapy [31].

Reinfection was thought to occur when virulent uropathogens gain entry into the urinary system or re-emerge from the bladder. Among the bacterial species, *E. coli* are responsible for 80–90% of community-acquired cases [31]. Other etiological agents reported are *Staphylococcus saprophyticus*, *Enterococcus faecalis*, *Klebsiella pneumoniae*, and *Proteus mirabilis* [12],[17]. The EQUC recovered more types of uropathogens that would have been missed from standard culture, an important consideration when half of these microbes were not found in women with UTI symptoms [19]. Molecular techniques allow a wider spectrum of bacterial genomic sequences to be detected, some of these are fastidious or uncultured species [14],[31]. As mentioned previously, a number of virulence factors are expressed to invade and colonize their host cells. Uropathogenic *E. coli* produce 2 types of fimbriae (or pili) that aim at specific receptors on the epithelial cells in the urinary system [31]. Furthermore, these pathogens may develop osmoadaptability to survive under the acidic environmental conditions [15].

There are evidences indicating a reservoir of pathogens may exist in the urinary system, possibly due to frequent failure of antibiotics to completely eradicate uropathogens, or the reemergence of the same organism resulting in recurrent infections [31]. While other microbes residing in the urogenital system are disrupted by antibiotics, pathogens may penetrate deeper to persist and re-infect the host. As *E. coli* population increases resulting in reinfection, the proportion of *Lactobacillus* that normally resides in the urogenital system notably decreases [31]. Two species are predominant in healthy women: *Lactobacillus (L.)* *crispatus* and *L. iners* [12],[18],[19],[20]. These organisms play an important role in maintaining the health of urinary tract as their metabolic byproducts (such as lactic acid, hydrogen peroxide) interfere with the adhesion of *E. coli* by downregulating some virulence factor expressions [5],[15],[31]. These results prompt replenishing lactobacilli as a strategy to manage recurrent UTIs, and probiotics have been considered due to promising results in maintaining a balanced microbiome in other physiological systems [38]. However, several clinical trials compared the effectiveness of probiotics versus antibiotics with inconclusive results [30]. Nevertheless, the general consensus is probiotic has merits over long term antibiotic treatments, the current method of managing UTIs, and warrants more research in this area. One of the main advantages is the lack of collateral damage, i.e., no increase in resistance rate, when a non-antibiotic prophylaxis is used. Several reports indicated high incidences (up to 100%) of resistance even among asymptomatic individuals when antibiotics were administered [5],[30],[31]. In some cases, persistence remained even after completion of the course [31].

### 3.2. Urgency urine incontinence

Urgency urinary incontinence (UUI) is a chronic condition that accounts for 1.5–22% of the US population, mostly affecting women and the elderly [16],[20]. Although a proportion of cases resolve after some time, it poses a significant financial burden costing over $76 billion in 2015 [18].

In spite of its prevalence, UUI is a poorly understood urinary condition often attributed to abnormal neuromuscular signaling or functioning [18],[20]. Because the symptoms seem to overlap with UTI, it is plausible that microbiome also plays a significant role in UUI [16],[18],[25]. A prospective cohort study using both culturing (EQUC) and molecular (16S rRNA gene sequencing) methods showed more diversity in women affected by UUI [20]. With combined methods, others reported genera such as *Actinobaculum, Actinomyces, Aerococcus, Arthrobacter*, and *Oligella*; many of which contain emerging uropathgoen members (e.g., *Aeroccocus urinae, Oligella ureolytica*) [11],[18] There seems to be a correlation between symptom severity and microbial diversity, as a study using 16S rRNA gene sequencing found increased UUI symptom gravity associated with lower microbial types [25]. In several studies, both women with and without UUI showed *Lactobacillus* and *Gardnerella* as the two most dominant genera by molecular techniques; the former plays a protective role as mentioned above, but the latter often associated with bacterial vaginosis [4],[14],[23]. For *Lactobacillus* there is additional distinction at the species level—*gasseri* was more frequent from UUI samples but *crispatus* more readily encountered from the control [18]. It is unclear how these two species differ in their role in the urinary system. Additional genera recovered by molecular (16S rRNA) and culturing (EQUC) methods in the urinary microbiome are *Bifidobacterium, Enterococcus, Actinomyces, Prevotella* and *Atopobium*. These are also present in vaginal tracts which suggest the possibility of a shared urogenital microbiome [14],[18]. The protective role of urine microbiome in the bladder and how they influence UUI warrant further studies.

### 3.3. Urolithiasis

The development of calcium oxalate stones, or urolithiasis, is one of the most common disorders of the urinary tract. It affects 8.8% of the population in the US, and over 300,000 people go to emergency room for such problem each year [39]. Calcium oxalate seems to be the most common type; specifically, oxalate has been reported in 75% of the urinary stones [33]. Oxalate is produced from amino acid metabolism; because it is toxic, it must be excreted by the kidney [33],[40]. Epidemiological studies and animal models indicate a correlation between the oxalate excretion and kidney stone development; thus, decreasing dietary oxalate and calcium intake would likely reduce such risk [34].

There have been several postulates regarding the interactions of microbiome and stone formation. One of the associations involved a bacterium, *Oxalobacter (O.) formigenes*, which was identified with a possible role in reducing recurrent calcium oxalate kidney stone formation [33]. As an anaerobic organism residing in human's intestine, it uses oxalate as main carbon and energy sources [40]. In fact, this bacterium degrades approximately 51% of the dietary oxalate per day and regulates the net intestinal transport of oxalate [41]. Consequently *O. formigenes* lowers the oxalate concentration for absorption in the colon which in turn decreases the amount excreted from the kidney [33]. As a case in point, colonization of *O. formigenes* resulted in 70% decrease in the risk of recurrent stone formation [5],[34].

Previously, *O. formigenes* was mostly detected by anaerobic culturing, a time consuming method with varying results. The advancement in molecular techniques (genome sequencing and 16S rRNA gene sequencing) enable more precise assessment of this organism in the microbiome. In the US, the prevalence of *O. formigenes* is relatively low (31% of population) in adults, perhaps stemmed from common antibiotic usage eliminating this bacterium from the gut [5],[40]. This was supported by the absence of *O. formigenes* in women with recurrent UTI, their frequent antibiotic treatments likely attributed to the increase oxalate excretion. On the other hand, this bacterial population is stable for a period of time (at least up to six months) once colonized. Diets with high oxalate can induce *O. formigenes*, and there is an interplay between dietary calcium, oxalate and the colonization of *O. formigenes* that result in urinary oxalate level of an individual [33],[34]. Other organisms (e.g., species of *Lactobacillus*) have also been implicated in degrading oxalate. However, they are not as efficient as *O. formigenes* so they will not be further discussed.

Another interaction between microbiome and kidney stone was reported by Barr-Beare et al [42]. Although this study only involved 5 young patients, 16S rRNA sequencing and EQUC methods showed the kidney stones contained multiple bacterial taxa. The dominant type (80%) was *Enterobacteriaceae*, leading the authors to suggest possible association of this bacterial family with pediatric kidney stones. Furthermore, individuals with stones often have higher incidences of UTI. How this observation affects future treatments of both hypercalciuria (high calcium in urine) and UTI awaits additional studies.

### 3.4. Bladder cancer

Urothelial carcinoma, mostly affecting the bladder, is one of the most common cancers in the urinary system [43]. It accounted for approximately 77,000 new cases with more than 16,000 deaths in 2016 [44]. Among the risk factors is chronic urinary tract infections especially in the bladder. Because microbiota have been involved in cancers of other organs, it is conceivable that they may play a role in urothelial carcinoma [5],[43].

One such study utilizing sequencing technology, albeit with a small sample size, showed the average genera found in cancer patients were noticeably higher than those healthy individuals (73.1 ± 42.8 versus 42.6 ± 19.4) [43]. *Acinetobacter* was the predominant genus and *Streptococcus* population was considerably elevated in cancer samples. These results indicate possible association between altered microbiota, both in population types and numbers, and urothelial carcinoma.

On the other hand, microbiota in the bladder may be involved in the prevention of recurrent superficial bladder cancer [5]. Some evidences indicated *L. casei* could reduce superficial bladder cancer recurrence, leading to considerations in applying probiotics as an alternative therapy [5]. The mechanism of action and interactions of lactobacilli with different microbial populations, however, remain as knowledge gaps.

## 4. Conclusion

Assessing microbiome in the urinary system has been a recent development, after expanded culturing technique and molecular analyses disputed the traditional thinking that urine was sterile. This review provided an overview of urinary microbiome and highlighted microbial members play an important role in urinary health of individuals. Understanding urinary microbiome, therefore, allows a more holistic approach in the diagnosis, treatment, and prevention of diseases and disorders in this system [17],[20].

There are various gaps that await further studies in urinary microbiome; such as mechanism of colonization (both beneficial organisms and uropathogens), predisposition for disorders due to certain microbial species, and the interplay between diets, probiotics, and other microorganisms. Because of its proximity to other physiological niches, the association between urinary microbes and other microbiomes (gut, genital) may enable a comprehensive assessment of certain diseases (e.g., kidney stone, chronic kidney diseases, bladder cancer) [5],[43]. An example is how carcinogens produced in the gut, filtered by the kidneys, and stored in the bladder prior to excretion influence urinary microbiota. A better understanding in these areas will aid in effective and more precise treatments in the future.

Most of the studies cited in this review were conducted with 16S rRNA gene sequencing as their molecular analysis method. Although powerful, its utility in comprehensive assessments of microbiome has certain limitations. For incidence, primers designed from this genetic region specifically target bacteria and at times only capable of genus level identification [10]. On the other hand, whole genome sequencing and metagenomics analyses are highly sensitive in detecting organisms present in individuals. This was demonstrated by the study of Barnett et al. when assessing *O. formigenes* in healthy young adults [34]. Analyzing the complete genome content of a sample is also broader in scope and allows distinguishing members of the entire microbial communities including viruses, fungi, and parasites. It is even capable of detecting species that are low in numbers which may be overlooked by other molecular methods. Furthermore, the genomic data facilitate studies of genetic composition to understand how organisms respond to different environments (e.g., antibiotics, stress) and their mechanisms to colonize the urinary systems [41]. Next generation sequencing technology and deep sequencing will enable the elucidation of core microbial populations and ascertain their roles in the urinary tract system [43]. This information potentially could lead to early indicators for diseases and the development of diagnostic tests.

Urinary microbiome research comes at the right time as a National Microbiome Initiative announced in 2016 aims at promoting integrated microbiome studies across different ecosystems by engaging interdisciplinary teams [45]. The Initiative also mentioned new platform technologies, among them is whole genomic sequencing which continues to advance significantly and its cost reduced considerably in recent years. This technology is no longer limited to big research and commercial laboratories. Bioinformatics capability has also expanded substantially to address the emergent needs of processing large amount of sequencing data and performing analysis in a timely manner. Numerous tools are being developed with many available as open source for use in the community. Opportunities and potential are plentiful for future studies in urinary microbiome.
